# The Pathological Role of LDL in Membranous Nephropathy and Diabetic Nephropathy and the Protective Efficacy of LDL Apheresis: A Narrative Review

**DOI:** 10.3390/toxins18010029

**Published:** 2026-01-08

**Authors:** Goh Kodama, Kensei Taguchi, Yusei Wada, Kaoru Nakano, Ryo Shibata, Kei Fukami

**Affiliations:** 1 Division of Nephrology, Department of Medicine, Kurume University School of Medicine, Kurume 830-0011, Japan; 2 Research Institute of Medical Mass Spectrometry, Kurume University School of Medicine, Kurume 830-0011, Japan

**Keywords:** LDL apheresis, membranous nephropathy, diabetic nephropathy

## Abstract

Diabetic nephropathy (DN) is the leading cause of end-stage kidney disease worldwide. One-third of patients with DN develop primary glomerulonephritis, and membranous nephropathy (MN) is the most common concurrent glomerulonephritis. Nephrotic syndrome (NS) due to DN and MN is often refractory to immunosuppressants because increased levels of low-density lipoprotein (LDL) not only accelerates kidney injury but also reduce the bioavailability of cyclosporine, a first-line immunosuppressant for MN. Given the pathological role of LDL, especially oxidized LDL, reducing LDL cholesterol levels can help achieve remission of NS and halt the progression of kidney injury. Although some lipoproteins are not excreted by the kidneys, excessive LDL, including oxidized LDL, can be considered uremic toxic-like factors that contribute to the development of NS or DN. We encountered a 74-year-old patient with concomitant DN and MN who achieved complete remission following additional LDL apheresis (LDL-A) with immunosuppressant therapy. Here, we provide a narrative review summarizing the role of LDL, especially ox-LDL, in the progression of DN and glomerulonephritis, including MN, and discuss the therapeutic rationale for LDL-A. We also present a representative case of concomitant MN and DN refractory to conventional immunosuppression who achieved clinical improvement following LDL-A.

## 1. Introduction

Diabetic nephropathy (DN) is the leading cause of end-stage kidney disease (ESKD) worldwide and is strongly linked to excess mortality and a high risk of cardiovascular disease [1]. The International Diabetes Federation anticipates that the global number of patients with diabetes will rise to 784 million by 2045 [2]. Considering that approximately half of patients with type 2 diabetes develop DN, the disease burden is expected to grow in parallel. As aging societies progress, heterogeneous kidney injury driven not only by hyperglycemia but also by hyperlipidemia, hypertension, and aging has gained acceptance worldwide. Moreover, concurrent glomerular pathologies such as membranous nephropathy (MN), IgA nephropathy, and nephrosclerosis frequently overlap with DN, further complicating diagnosis and treatment.

MN is a representative cause of nephrotic syndrome (NS) in adults and older individuals. One-third of MN patients achieve spontaneous remission, yet a comparable proportion progress to persistent kidney dysfunction within 15 years, even when treated with immunosuppressants [3]. In fact, complete remission was attained in only 67.8% of adult patients with MN, and the rate further fell to 53% among patients aged over 65 years in the Japanese Nephrotic Syndrome Cohort Study (JNSCS) [4]. Several guidelines discourage glucocorticoids (GCs) monotherapy; instead, combination regimens with immunosuppressants, such as cyclosporine, are associated with better renal outcomes. Nonetheless, a substantial subset of patients in clinical practice still requires longitudinal glucocorticoid exposure, which can exacerbate glycemic control in MN complicated by diabetes.

Dyslipidemia, characterized by increases in total cholesterol (TC) and low-density lipoprotein (LDL) cholesterol, is frequently observed in NS due to increased production of very low-density lipoprotein (VLDL) in response to hypoalbuminemia. Pharmacologically lowering of serum LDL cholesterol levels does not reduce proteinuria in patients with NS [5]. However, LDL apheresis (LDL-A), an extracorporeal therapy that selectively removes circulating LDL cholesterol, can reduce albuminuria and slow down the progression of kidney dysfunction in DN, while being long established for homozygous familial hypercholesterolemia and severe peripheral arterial disease. Moreover, with declining renal function, oxidized LDL (ox-LDL) increases and plays a pivotal role in the progression of kidney injury. The reno-protective effects of LDL-A may reflect the simultaneous clearance of immune complexes, oxidized lipoproteins, and pro-inflammatory cytokines. Here, we summarize the role of LDL, especially ox-LDL, in the progression of DN and MN in this narrative review and demonstrate the therapeutic potential of LDL-A by presenting a representative case of a 74-year-old patient of concomitant MN and DN refractory to conventional immunosuppression who achieved marked clinical remission following LDL-A.

## 2. Ox-LDL-Induced Inflammation Are Involved in the Progression of DN

Uremic toxins are defined as accumulated solutes that rise with declining kidney function, are normally excreted by the kidneys, and interact negatively with biological processes. Although some lipids and lipoproteins are not excreted into urine under normal conditions, lipid metabolism changes in the context of CKD, and their modifications alter biological functions, promoting further progression of CKD. Ox-LDL is generated by oxidative stress through enzymatic and non-enzymatic pathways, and its accumulation has been observed in CKD in previous clinical studies [6]. High levels of immune complexes containing ox-LDL are also associated with macroalbuminuria in type 1 diabetes [7]. Ox-LDL not only activates atherogenic pathways but also potentiates inflammation, tubular injury, and kidney fibrosis via activation of angiotensin II, CD36, and the receptor for advanced glycation end products (RAGEs) [8,9,10]. Oxidative stress increases as renal function declines, promoting the generation of ox-LDL. Ox-LDL then accelerates kidney injury, creating a positive feedback loop. Accordingly, ox-LDL may be classified as a broad category of toxins related to CKD.

## 3. Aberrant Inflammation Contributes to the Progression of DN

Glomerular hyperfiltration initiates microalbuminuria, which progressively advances to overt proteinuria and a decline in kidney function, accompanied by nodular glomerulosclerosis namely Kimmelstiel–Wilson lesions, and extensive tubulointerstitial fibrosis. DN progresses to ESKD within 10–15 years without therapeutic intervention. Accordingly, optimizing glycemic control with glucose-lowering agents such as metformin [11], controlling blood pressure with renin–angiotensin system (RAS) blockade [12], and using sodium–glucose cotransporter 2 (SGLT2) inhibitors [13] and GLP-1 receptor agonists [14] have been shown to slow disease progression. Recent clinical trials clearly showed that the blockade of mineralocorticoid receptor with finerenone significantly prevents the progression of DN [15]. Furthermore, the combination of MR blockade and SGLT2 inhibition exerts even greater reno-protection in patients with type 2 diabetes [16]. Although such intensive treatments are linked to a reduced number of patients with diabetes requiring dialysis initiation in Japan [17], a subset of patients with overt proteinuria remains at high risk for progression to ESKD.

At the molecular level, aberrant inflammation has emerged as a robust contributor to the progression of DN in addition to classical pathological factors, including the protein kinase C pathway [18], oxidative stress [19], and AGEs [20]. C-X-C chemokine ligand 16 (CXCL16) exists as transmembrane and soluble isoforms. The transmembrane form acts as a scavenger receptor for ox-LDL in macrophages, promoting atherosclerosis. By contrast, soluble CXCL16 is generated by ADAM10-mediated shedding and binds to the C-X-C motif chemokine receptor 6, thereby promoting inflammation. Thus, targeting CXCL16 attenuates glomerular injury and kidney fibrosis [21]. Soluble tissue necrotic factor (TNF) receptors 1 and 2 serve as biological markers for predict progression to ESKD in patients with diabetes [22]. Complement activation, including C4d and the membrane attack complex (MAC), has been observed within kidney intrinsic cells and positively correlates with the histological severity of DN [23]. C5a is also a pathological driver of increased oxidative stress, leukocyte recruitment, and tubular injury in the context of DN [24,25]. Pentoxifylline is a pharmaceutical agent classified as a xanthine derivative that has inhibitory properties against TNF-mediated inflammatory signaling. A recent randomized trial showed that albuminuria and the eGFR slope were significantly attenuated by administration with pentoxifylline [26]. The C-C chemokine receptor type 2 antagonist CCX140-B reduced albuminuria by suppressing RAS activation in patients with DN [27].

## 4. Ox-LDL and Excess LDL Interferes with Therapeutic Actions of Immunosuppressants Against MN

MN is an autoimmune glomerular disease in which subepithelial immune complexes form in situ on the podocyte surface, triggering complement activation. The level of proteinuria at onset is associated with long-term kidney outcomes [28]. Since the discovery of anti-phospholipase A2 receptor (PLA2R) antibodies in 2009, several investigations have identified specific antigens to clarify disease etiology and activity [29]. For instance, thrombospondin type-1 domain-containing 7A (THSD7A) is responsible for idiopathic MN, and positivity for neural epidermal growth factor-like 1 (NELL-1) is linked to malignancy-induced MN. Autoantibodies binds to specific antigens expressed on the surface of podocytes, which induces subepithelial immunocomplex deposition [30]. These immunocomplexes consistently drive complement activation, including MAC (C5b-9) formation, cytoskeletal disorganization, and oxidative stress production, resulting in podocyte injury. Furthermore, it has been shown that Ox-LDL downregulates integrin α3 and increases fibronectin and reactive oxygen species production in cultured podocytes [31], and long-term exposure of podocytes to massive proteinuria enhances the uptake of ox-LDL, resulting in increased pro-inflammatory cytokine production.

According to the Japanese Society of Nephrology (JSN) clinical guidelines, oral glucocorticoids (GCs) monotherapy is recommended as first-line therapy for primary MN with nephrotic-range proteinuria [32]. For GC-resistant or relapsing MN, the addition of other immunosuppressive agents, such as the calcineurin inhibitor cyclosporine, the alkaline agent cyclophosphamide, or the purine synthesis inhibitor mizoribine, to GCs confers anti-proteinuria effects [33]. Cyclosporine is a first-line additive therapy to GCs because of its low incidence of adverse effects. Cyclosporine is a lipophilic peptide, with approximately 50% bound to circulating lipoproteins. In vitro studies have demonstrated that cyclosporine exhibits the greatest affinity for LDL, compared with VLDL and high-density lipoprotein (HDL). Consequently, LDL serves as a carrier for cyclosporine into T lymphocytes via LDL receptors (LDL-Rs) [34]. In the context of hypercholesterolemia, LDL-R might be saturated with large amounts of LDL, which inhibit the adsorption of cyclosporine. Thus, excess LDL or ox-LDL alters the bioavailability of cyclosporine, interfering with MN remission. Therefore, rapid removal of LDL by LDL-A presumably recovers cyclosporine uptake into T lymphocytes through rebooting LDL-R function [35].

## 5. LDL-A Confers Reno-Protective Efficacy in a Wide Range of Glomerulonephritis, Including DN

Among primary glomerulonephritis, LDL-A was initially approved for focal segmental glomerulosclerosis (FSGS). In LDL-A using a dextran sulfate column (Liposorber, Kaneka), which is prepared by coating porous Sepharose beads with dextran sulfate, electrostatic interactions between negatively charged dextran sulfate and positively charged apoprotein B on the surface of lipoproteins achieve adsorption of LDL. A multi-center clinical trial, the POLARIS study, demonstrated the reno-protective efficacy of LDL-A in patients with refractory NS owing not only to FSGS but also to minimal change disease (MCD) and MN [36]. The average number of LDL-A sessions and the volume of treated plasma were 9.6 ± 2.7 sessions and 3.5 ± 0.8 L, respectively. Thereafter, LDL-A treatment for refractory NS, including MCD and MN was approved in Japan in June 2024. We have summarized case series indicating that LDL-A is a possible therapeutic option to treat primary glomerulonephritis, including FSGS (Table 1), MCD (Table 2), and MN (Table 3). Details of the literature search strategy and study selection are provided in the “Section 6”.

In the context of DN, a nationwide prospective study in Japan reported that six-month treatment with LDL-A improved all-cause mortality and reduced the levels of proteinuria in patients with diabetes who had massive proteinuria and hypercholesterolemia [57]. On the basis of previous clinical study outcomes, LDL-A has been approved for insurance coverage in Japan for DN patients with massive proteinuria and refractory hypercholesterolemia since 2022. In contrast, in the United States and Europe, LDL-A is not currently an approved indication for DN; rather, it is utilized as an extracorporeal lipid-lowering therapy for severe familial hypercholesterolemia [58,59]. Meanwhile, the American Society for Apheresis recommends its use for FSGS-related nephrotic syndrome [60]. Recent case series revealed that significant reduction in proteinuria and long-term remission was achieved with LDL-A in diabetic patients with refractory NS [36]. Beyond lowering serum levels of LDL, the procedure removes humoral permeability factors, pro-inflammatory cytokines, and pro-coagulant molecules implicated in glomerular injury. Interleukin (IL)-1α, IL-4, IL-6, IL-10, TNF-α, vascular endothelial growth factor, and p-selectin were significantly reduced by LDL-A using Liposorber [61,62]. CXCL16, a pro-inflammatory cytokine that interacts with ox-LDL, possesses a positive charge and has been shown to bind to dextran sulfate because of electrostatic properties [63], suggesting that LDL-A using dextran sulfate cellulose can absorb CXCL16 from the bloodstream, thereby inhibiting the pathological role of ox-LDL and inflammation. The removal of ox-LDL with LDL-A has been shown to attenuate podocyte injury via modulating inflammation in the kidneys [64]. Collectively, accumulating evidence has proven the reno-protective efficacy of LDL-A across diverse kidney diseases.

## 6. Literature Search and Selection

We performed a PubMed-based literature search for reports of LDL apheresis in FSGS, MCD, and MN using disease-specific keywords combined with “LDL apheresis” (e.g., “focal segmental glomerulosclerosis” AND “LDL apheresis”). Studies without accessible full text were excluded, and eligible reports are summarized in tables.

## 7. Representative Case Description

A 74-year-old male had been diagnosed with type 2 diabetes three years ago, followed by the initiation of oral hypoglycemic medications, such as canagliflozin, teneligliptin, and metformin, which achieved optimal glycemic control with HbA1c levels of 6–7%. At this timepoint, blood urea nitrogen (BUN) and serum levels of creatinine (Cr) were 14.6 and 0.65 mg/dL, respectively. Two years later, urinalysis first revealed proteinuria (1+). Over the next few months, serum creatinine increased from 0.88 to 1.11 mg/dL, while serum albumin decreased from 3.9 to 1.9 g/dL. Two months later, the ratio of urinary protein per urinary Cr (UP/UCr) increased to 21.63 g/gCr, while serum albumin further declined to 1.5 g/dL, both of which met the criteria for NS. He was referred to a primary physician clinic, where the third-generation mineralocorticoid receptor esaxerenone and the angiotensin receptor–neprilysin inhibitor sacubitril/valsartan were initiated. Within a month, exertional dyspnea appeared, leading to initiation of intravenous administration with furosemide; however, severe edema in lower extremities did not improve, and kidney dysfunction progressed, with serum Cre of 1.61 mg/dL. For further investigation, he was referred to the Division of Nephrology, Kurume University Hospital.

**Figure 1:**
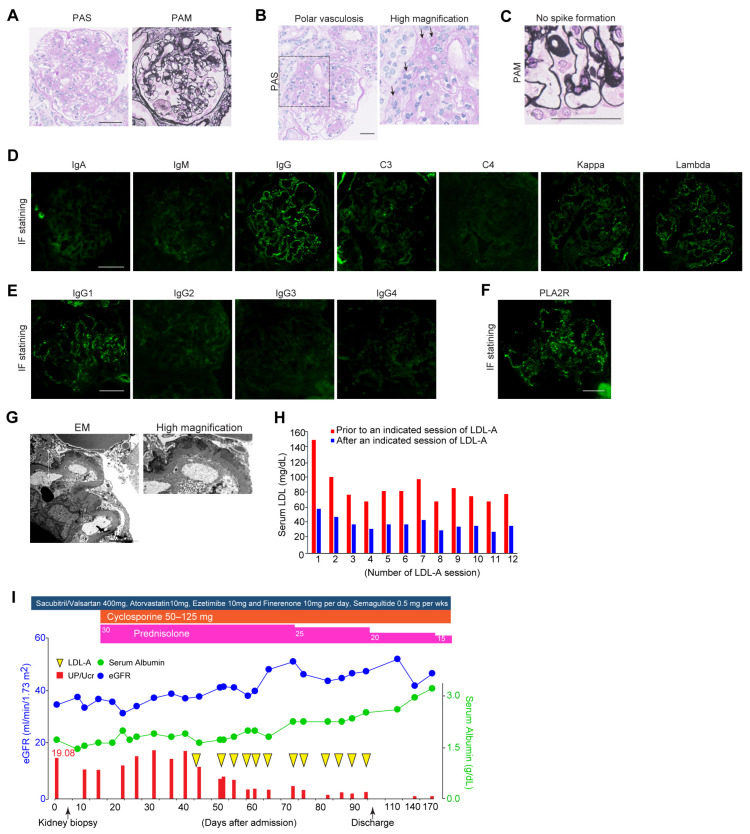
Clinical course and manifestations of the present case with DN and MN treated with LDL-A. (**A**) Representative images of Kimmelstiel–Wilson nodular lesions on PAS and PAM staining. Scale bar: 50 µm. (**B**) Representative images of polar vasculosis on PAS staining. Scale bar: 50 µm. Arrows; polar vasculosis. (**C**) No spike formation on PAM staining. Scale bar: 50 µm. (**D**) Immunofluorescence staining for immunoglobulins and light chain deposition along the GBM. Scale bar: 50 µm. (**E**) Immunofluorescence staining for IgG subclass deposition along the GBM. Scale bar: 50 µm. (**F**) Immunofluorescence staining for PLA2R deposition along the GBM. (**G**) Representative images of electron microscopy showing electron-dense deposits(EDD) along the GBM. Arrows; EDD. Scale bar: 2 µm. (**H**) Serum LDL levels before and after a session of LDL-A. (**I**) Clinical course of the present case before and after LDL-A, such as glucocorticoids and cyclophosphamide. The blue line indicates serum levels of albumin (g/dL) and the green line indicates estimated GFR (mL/min/1.73 m^2^). Red bars indicate the levels of proteinuria (UP/UCr). Magenta squares indicate the dose of prednisolone and orange squares indicate the dose of cyclophosphamide. PAS, periodic acid–Schiff; PAM, periodic acid–methenamine silver; GBM, glomerular basement membrane; LDL, low-density lipoprotein; LDL-A, LDL apheresis. UP/UCr, ratio of urine protein per urine creatinine.

At admission, pleural effusion was detected by chest X-ray examination, and severe leg edema was pointed out. Although we hypothesized DN-related NS, no signs of diabetic retinopathy were detected on fundus examination. The fasting triglyceride level was 203 mg/dL, and serum levels of LDL and HDL were 116 and 60 (mg/dL), respectively. Serological results showed slight elevation in immunoglobulins (IgG: 1782 and IgM: 201 (mg/dL)); meanwhile, no abnormalities in complement levels and autoimmune antibodies were detected. Up/UCr remained at 19.08 g/gCr, with a low selectivity index of 0.37 and urinary sediment including fatty and granular casts. As the etiology of NS remained unclear, a kidney biopsy was performed. Kidney biopsy showed Kimmelstiel–Wilson nodules in almost all glomeruli (Figure 1A). Mesangiolysis and exudative lesions were detected in approximately 30% of the glomeruli, and polar vasculosis was found (Figure 1A,B). No spikes along the glomerular basement membrane (GBM) were identified in periodic acid–methenamine silver (PAM) staining (Figure 1C). Immunofluorescence (IF) staining demonstrated granular positivity for IgG (mainly IgG1), C3, light chains of kappa and lambda, and PLA2R along the GBM in almost all glomeruli (Figure 1D–F). Electron microscopy (EM) revealed the presence of electron-dense deposits (EDDs) located in the subepithelial region of the GBM, which was classified as stage I according to the Ehrenreich–Churg classification (Figure 1G). These findings led a diagnosis of DN complicated by early-MN.

Considering the diagnosis, the dosage of sacubitril/valsartan was increased from 200 to 400 mg per day, esaxerenone was switched to finerenone, and semaglutide, a glucagon-like peptide-1 receptor agonist, was initiated. For the treatment of MN, GCs at 30 mg per day were started in combination with cyclosporine at 100 mg per day. Despite four weeks of the abovementioned intensive care, no improvement in NS was observed. Furthermore, serum levels of LDL increased to 153 mg/dL despite treatment with atorvastatin at 10 mg per day for several months. Given that increased LDL interferes with the effects of immunosuppressant therapy, we decided to initiate LDL-A as an additive therapy. LDL-A was performed twice weekly for a total of twelve sessions. The blood purification system used was the MA-03 device^®^ and plasma separation was achieved using the primary membrane OP-05D^®^ for plasma filtration and the secondary membrane Liposorber LA-15^®^ for lipid adsorption. The processed plasma volume per session was approximately 2 L. Anti-coagulation was maintained with low-molecular-weight heparin, administered as an initial bolus of 2000 units followed by continuous infusion at a rate of 1000 units per hour. The average LDL removal rate per LDL-A session was approximately 53% (Figure 1H), and serum LDL levels were reduced from 153 mg/dL to 37 mg/dL after completion of the 12th session. The average removal rate of LDL at each session was calculated as 55.3 ± 4.2%. LDL-A resulted in a marked reduction in proteinuria and recovery of kidney function. UP/UCr decreased from 19.08 to 4.07 g/gCr, and the estimated glomerular filtration rate increased from 35 to 47 mL/min/1.73 m^2^, with an improvement in serum albumin from 1.1 to 3.0 g/dL (Figure 1I). Over the following year, GCs were tapered to 7.5 mg/day without deterioration in glycemic control. Atorvastatin at 10 mg per day was continued to maintain LDL cholesterol at the lowest achievable level.

## 8. Discussion

Ox-LDL and increased levels of LDL induce aberrant inflammation, tubular damage, and kidney fibrosis, leading to the progression of DN and glomerulonephritis. Since recent evidence has revealed the protective efficacy of LDL-A, LDL-A became an approved therapeutic option authorized by the Pharmaceuticals and Medical Devices Agency (PMDA) for DN in 2022 [57] and for NS, including MN and MCD, in 2024. Accumulating case series indicate that LDL-A is a therapeutic option for treating primary glomerulonephritis, including FSGS (Table 1), MCD (Table 2), and MN (Table 3). Our patient with type 2 diabetes developed NS due to concomitant primary MN, and the massive proteinuria was resistant to immunosuppressant therapy and sustained; thus, additional LDL-A was required. A total of twelve sessions of LDL-A reduced proteinuria, with substantial reduction in biochemical parameters, including hypercholesteremia. This is a narrative review originated from descriptive case report of DN and MN in which LDL-A was followed by clinical improvement. However, current clinical evidence is derived from uncontrolled observations and does not include comparator groups; therefore, robust conclusions regarding efficacy cannot be drawn. Nonetheless, the collective findings suggest that LDL-A may be associated with reduced proteinuria and recovery of kidney function in selected patients.

DN is known to solely induce massive proteinuria; however, the concomitant existence of primary glomerulonephritis is not rare. Previous clinical observations of patients with DN who underwent kidney biopsies demonstrated that 27.4% showed concurrent existence of primary glomerulonephritis [65]. MN was the most common concurrent primary GN, followed by IgA nephropathy and arterio-nephrosclerosis [65]. Since diabetic patients are at higher risk of malignancy compared to non-diabetic individuals, progressive proteinuria may be an important clinical manifestation, indicating malignancy-related secondary MN. Accordingly, longitudinal monitoring of proteinuria is warranted not only to assess the progression of DN but also to determine the concurrent development of primary glomerulonephritis, which necessitates different intensive care from those for DN. Furthermore, the absence of diabetic retinopathy still provides clinical insights for detecting the presence of primary GN in patients with T2DM, because the frequency of retinopathy is completely different between patients with DN alone and those with DN plus primary GN (6.5% vs. 32.8%). Therefore, further evaluation might be required if diabetic patients develop unexplainable progression of massive proteinuria.

Ox-LDL has been reported to accumulate in podocytes of patients with DN and MN [66]. CXCL16, known to bind to ox-LDL, is recruited to and accumulates in podocytes in MN and DN, where it acts as a scavenger receptor for ox-LDL, leading to inflammation in podocytes [67]. Considering that ox-LDL deposition plays a pivotal role in podocyte injury, removal of ox-LDL by LDL-A is supposed to inhibit MN-related proteinuria. This concept is supported by experimental evidence showing that statins protected podocytes exposed to ox-LDL [68]. Furthermore, LDL-A may improve the response to cyclosporine via modulating LDL-related drug delivery. As a result, marked improvement in proteinuria can permit smooth tapering of GCs, thereby reducing the risk of GC-related adverse effects. A major recent concern among nephrologists is infectious diseases in elderlies with NS, which is directly linked to mortality [69]. In addition to the positive correlation between GC dose and the risk of serious infection [70], patients with T2DM are normally susceptible to infectious diseases [71]. LDL-A is generally considered a well-tolerated procedure. Immunoglobulins (Igs) are not removed by LDL-A due to their molecular weights of; thus, LDL-A itself is considered not associated with infectious diseases. Therefore, LDL-A is not only supposed to promote remission of MN and DN but also to reduce the doses of GC and immunosuppressants, thereby contributing to avoidance of the risk of death by serious infections. Elderly accounted for a considerable proportion of first-time native renal biopsy cases in Japan, most notably those in their seventies [72]. Elderlies are more likely to develop MN, whereas IgA nephropathy is more common in younger patients [73]. The oldest patient enrolled in the POLARIS study was 87 years old, and our case was 74 years old, indicating that additional LDL-A may be well-tolerated in elderlies.

Future studies are warranted to elucidate the long-term reno-protective effects of LDL-A in patients with DN complicated by concurrent immune-mediated glomerular disease. In particular, prospective studies examining its role in optimizing immunosuppressive drug delivery and improving metabolic control would further clarify its clinical utility. Identification of predictive biomarkers for treatment response, such as ox-LDL, may also enable better patient selection and more individualized therapeutic strategies, including LDL-A.

## 9. Conclusions

The present review highlights potential pathways through which ox-LDL accumulation and ox-LDL-related inflammatory signaling contribute to podocyte injury in DN and glomerulonephritis, providing a rationale for extracorporeal removal of LDL and ox-LDL. Excess LDL and ox-LDL may interfere with cyclosporine pharmacodynamics via LDL-dependent drug transport, thereby attenuating responsiveness. In this context, our case of concomitant DN and MN suggests that LDL-A may be associated with improvement in NS and may facilitate GC tapering under ongoing cyclosporine therapy. These observations support the potential benefit of LDL-A and warrant further prospective investigation, although they do not permit causal inference. Future prospective studies with longer follow-up and appropriate comparator groups are warranted to validate the efficacy of LDL-A and to evaluate its impact on long-term kidney outcomes.

## Figures and Tables

**Table 1:** Protective efficacy of LDL-A for FSGS.

Author (Year)	Age/Sex	Toal Session Number (*n*)	Initial Therapy with IS Prior to LDL-A	Required Dose of IS Following LDL-A	Clinical Outcome
Masutani et al. (2005) [37]	45/M	12	mPSL 500 mg × 3, oral mPSL16 mg, MMF 50 mg, and TAC 6 mg	mPSL12 mg, MMF 50 mg, and TAC 6 mg	PU decreased from 6.8 to 2.0 g/day (PR).
Miyazono et al. (2008) [38]	73/F	6	mPSL 500 mg × 3, GC 60 mg, CsA 150 mg, and required HD	GC 7.5 mg and CsA 75 mg	PU decreased from 7.6 to 2.3 g/day (PR). HD was discontinued.
Miura et al. (2009) [39]	61/M	4	GC 50 mg and required HD	GC 40 mg	PU completely disappeared (CR). HD was discontinued.
Araki et al. (2015) [40]	43/F	12	GC 40 mg and required HD	GC 30 mg	PU reduced to 1.1 g/day. HD was discontinued.
Shima et al. (2022) [41]	39/F	12	mPSL 500 mg × 3, GC 40 mg, CsA 100 mg, and required HD	GC 20 mg and CsA 100 mg	PU decreased and CR was achieved.
Shima et al. (2024), Case 1–3 [42]	52/F, 63/M, 79/F	12	mPSL 500–1000 mg × 6, GC 30–50 mg, and CsA 150 mg	GC 20 mg with or without CsA 175 mg	PU decreased to 0.18–0.56 g/g Cr, consistent with CR.

Abbreviation: FSGS, focal segmental glomerulosclerosis; mPSL, methylprednisolone; GC, glucocorticoid; CsA, cyclosporine; MMF, mycophenolate mofetil; TAC, tacrolimus; IS, immunosuppressant; HD, hemodialysis; PU, proteinuria; CR, complete remission; PR, partial remission.

**Table 2:** Protective efficacy of LDL-A for MCD.

Author (Year)	Age/Sex	Toal Session Number (*n*)	Initial Therapy with IS Prior to LDL-A	Required Dose of IS Following LDL-A	Clinical Outcome
Okada et al. (1996) [43]	20/M	6	GC 40 mg and CsA 300 mg	GC 15 mg, CsA 300 mg	PU decreased from 12.6 to 0.96 g/day.
Stenvinkel et al. (2000) [44]	34/M 62/M	13	GC and CsA	GC and CsA discontinued	PU decreased.
Yoshizawa et al. (2003) [45]	50/F, 25/M, 45/F	12	GC	GC	CR was achieved in all 3 patients.
Kobayashi et al. (2006) [46]	17/M	4	GC 60 mg	GC discontinued	PU was reduced from 9.2 to 0.2 g/day.
Miyata et al. (2012) [47]	48/M	14	GC and other ISA.	GC and other ISA	CR was achieved.
Nakatani et al. (2018) [48]	48/F		mPSL 1000 mg × 3, GC and CsA 75 mg	GC continued and CsA 75 mg	PU decreased from 9.0 to 2.4 g/day.
Terada et al. (2020) [49]	49/F and 71/M	6	mPSL 500 mg × 3, GC 40 mg, and HD or PEX were required	GC 30–40 mg	CR was achieved in both cases. HD was discontinued.
Hiramatsu et al. (2025) [50]	75/F	7	mPSL 500 mg × 6, GC40 mg, and HD was required	GC 20 mg and CsA50 mg	PU reduced from 11.3 to 0.69 g/day at day 350. HD was discontinued.

Abbreviation: MCD, minor change disease; mPSL, methylprednisolone GC; glucocorticoid; CsA, cyclosporine; ISA, immunosuppressant; HD, hemodialysis; PU, proteinuria; PEX, plasma exchange; CR, complete remission.

**Table 3:** Protective efficacy of LDL-A for MN.

Author (Year)	Age/Sex	Toal Session Number (*n*)	Initial Therapy with IS Prior to LDL-A	Required Dose of IS Following LDL-A	Clinical Outcome
Ideura et al. (2000) [51]	46/M	12	mPSL 1000 mg × 3, GC 10 mg, CsA 150 mg, and AZA 50 mg	GC 10 mg, CsA 150 mg, and AZA 50 mg	PU was reduced from 9.0 to 0.5 g/day.
Sato et al. (2012) [52]	75/F, 35/M, 76/M	6	mPSL 500 mg × 6, GC 30–40 mg and CsA 150–200 mg	GC 12.5–25 mg and CsA 75–200 mg	PU was reduced from 4.06 to <0.3 g/day. CR was maintained for almost 2–8 years.
Yabuuchi et al. (2017) [53]	61/M	203	GC 60 mg and CsA 360 mg	GC40 mg and CsA discontinued	PU was reduced from 13.1 to <1 g/day.
Szymanski et al. (2019) [54]	49/F	11	mPSL, GC, rituximab, CsA, and CPA	GC continued while CsA and CPA discontinued	PU was reduced from 6.2 to 0.4 g/gCr.
Nishizawa et al. (2020) [55]	68/M	6	mPSL 1000 mg × 6 and GC 60 mg	Betamathasone 5 mg	PU was reduced from 6.26 to 2.9 g/gCr.
Nishizawa et al. (2022) [56]	39/M	12	GC 40 mg and CsA	GC 25 mg and CsA continued	PU was reduced from 10.6 to 2.3 g/gCr.

Abbreviation: MN, membranous nephropathy; mPSL, methylprednisolone; GC, glucocorticoid; CsA, cyclosporine; AZA, azathioprine; CPA, cyclophosphamide; IS, immunosuppressant; PU, proteinuria; CR, complete remission.

## Data Availability

No new data were created or analyzed in this study.
